# Pathophysiological Associations and Measurement Techniques of Red Blood Cell Deformability

**DOI:** 10.3390/bios15090566

**Published:** 2025-08-28

**Authors:** Minhui Liang, Dawei Ming, Jianwei Zhong, Choo Sheriel Shannon, William Rojas-Carabali, Kajal Agrawal, Ye Ai, Rupesh Agrawal

**Affiliations:** 1 School of Mechanical Engineering, Guangxi University, Nanning 530004, China; minhui@gxu.edu.cn (M.L.);; 2 Pillar of Engineering Product Development, Singapore University of Technology and Design, 8 Somapah Road, Singapore 487372, Singapore; 3 National Healthcare Group Eye Institute, Tan Tock Seng Hospital, Singapore 308433, Singapore; 4 Lee Kong Chian School of Medicine, Nanyang Technological University, Singapore 308232, Singapore

**Keywords:** red blood cell, deformability, biomedical research

## Abstract

Red blood cell (RBC), accounting for approximately 45% of total blood volume, are essential for oxygen delivery and carbon dioxide removal. Their unique biconcave morphology, high surface area-to-volume ratio, and remarkable deformability enable them to navigate microvessels narrower than their resting diameter, ensuring efficient microcirculation. RBC deformability is primarily determined by membrane viscoelasticity, cytoplasmic viscosity, and cell geometry, all of which can be altered under various physiological and pathological conditions. Reduced deformability is a hallmark of numerous diseases, including sickle cell disease, malaria, diabetes mellitus, sepsis, ischemia–reperfusion injury, and storage lesions in transfused blood. As these mechanical changes often precede overt clinical symptoms, RBC deformability is increasingly recognized as a sensitive biomarker for disease diagnosis, prognosis, and treatment monitoring. Over the past decades, diverse techniques have been developed to measure RBC deformability. These include single-cell methods such as micropipette aspiration, optical tweezers, atomic force microscopy, magnetic twisting cytometry, and quantitative phase imaging; bulk approaches like blood viscometry, ektacytometry, filtration assays, and erythrocyte sedimentation rate; and emerging microfluidic platforms capable of high-throughput, physiologically relevant measurements. Each method captures distinct aspects of RBC mechanics, offering unique advantages and limitations. This review synthesizes current knowledge on the pathophysiological significance of RBC deformability and the methods for its measurement. We discuss disease contexts in which deformability is altered, outline mechanical models describing RBC viscoelasticity, and provide a comparative analysis of measurement techniques. Our aim is to guide the selection of appropriate approaches for research and clinical applications, and to highlight opportunities for developing robust, clinically translatable diagnostic tools.

## 1. Introduction

Red blood cell (RBC) constitute approximately 45% of total blood volume and serve as the principal carriers of oxygen in the human body. Mature RBC are anucleate biconcave disks, typically 6.2–8.2 μm in diameter and 2–2.5 μm in thickness, with a mean corpuscular volume (MCV) around 92.8 femtoliters at a hematocrit of 42.5%. Their biochemical composition is approximately 19.5% water, 39.5% protein, 35.1% lipids, and 5.8% carbohydrates by weight. The absence of a nucleus and most organelles, coupled with reliance on anaerobic metabolism during their ~120-day lifespan [1], equips RBC with the flexibility required to traverse vessels ranging from 100 μm arterioles to 3 μm capillaries, thereby ensuring efficient microvascular perfusion [2].

**Figure 1:**
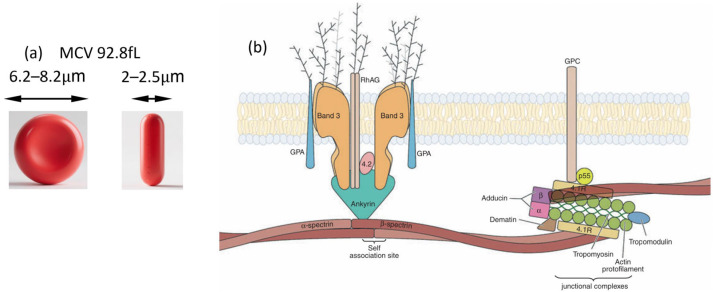
Schematic representation of RBC structure. (**a**) Morphology of normal RBC. (**b**) A schematic representation of components that consist of the RBC membrane. Figure adapted from Reference [5] with permission.

This exceptional deformability is determined by three key factors [3,4]: (1) the cell surface area-to-volume ratio, which influences shape and osmotic fragility; (2) cytoplasmic viscosity, largely governed by the mean corpuscular hemoglobin concentration (MCHC) and cell water content; and (3) the viscoelasticity of the membrane, determined by the spectrin network and lipid bilayer (Figure 1b) [5].

Together, these parameters dictate the mechanical response of RBC under shear stress and during passage through microvascular constrictions. Alterations in these properties occur in a wide range of physiological and pathological contexts, including sickle cell disease, malaria, diabetes mellitus, sepsis, ischemia–reperfusion injury, and storage lesions, with the disease-specific implications discussed in detail in Section 2.

Given the diagnostic and prognostic potential of RBC deformability, various approaches have been developed to characterize its mechanical properties [6]. These range from viscoelastic modeling frameworks, which provide fundamental insights into RBC mechanics (Section 3), to experimental measurement techniques spanning single-cell assays, bulk rheological methods, and emerging microfluidic platforms (Section 4).

In the past five years, most reviews of RBC deformability have been relatively narrow in scope—focused either on a single pathological context (e.g., aging [7] or diabetes [8]) or on specific measurement approaches (notably microfluidic platforms [9]); comprehensive reviews that span multiple disease states and compare traditional and emerging measurement strategies remain scarce.

Building on this gap, the present review provides a broader synthesis that integrates the biomedical significance of RBC deformability across diverse disease contexts, systematically compares conventional and emerging measurement techniques, and discusses key challenges and opportunities for clinical translation. Particular emphasis is placed on future directions, with the authors considering that approaches combining RBC imaging with artificial intelligence (AI) offer the most promising pathway toward high-throughput, reproducible, and clinically relevant deformability assessment (Section 5).

## 2. RBC Deformability in Health and Disease

The deformability of RBC is a critical element of cellular physiology. Any aberrations from this fundamental attribute may manifest in a wide array of pathophysiological conditions. These vary from shifts in normal physiological states—such as temperature, pH, osmotic pressure [10,11]—to severe pathological circumstances, inclusive of, but not limited to, prolonged RBC storage, sickle cell disease, malaria, diabetes mellitus, sepsis, and post-ischemic reperfusion injuries (summarized in Table 1).

The duration of RBC storage is a crucial determinant in assessing the quality of RBC for transfusion. As per the standard protocol, donor blood is typically stored at 4 °C for a period of 42 days. However, the rate of degradation in stored blood can vary among individuals, leading to disparate levels of hemoglobin functionality [27]. Decreased RBC deformability results from increased viscosity, primarily driven by the depletion of adenosine triphosphate (ATP) and accumulation of calcium levels [28]. To tackle this issue, previous studies suggest the use of RBC deformability as a simple, rapid, and cost-effective quality check for stored blood prior to transfusion. This measure could provide a reliable gauge of the storage quality of RBC and mitigate potential risks associated with transfusion [12,29,30].

Sickle cell disease is an autosomal recessive genetic disorder, characterized by the presence of abnormal sickle hemoglobin (HbS) in lieu of normal hemoglobin (HbA) [13]. The polymerization of HbS molecules into intracellular fibers during deoxygenation induces the trademark sickle cell shape. Sickle cell crises commonly correlate with elevated plasma viscosity, suggesting that fluctuations in this parameter may incite or exacerbate such crises [31]. Unlike healthy blood, which demonstrates a consistent decrease in velocity within small capillaries as plasma viscosity increases, the velocity of sickle blood exhibits irregular behavior [14], persisting even at considerably low plasma viscosities. This irregular behavior is perhaps due to alterations in cytokine profile and plasma protein concentration during crises. Increased levels of some plasma proteins, such as fibrinogen, may also cause a marked increase in RBC aggregation, leading to higher plasma viscosity at lower shear rates [32].

Malaria, particularly *Plasmodium falciparum* malaria, poses a lethal threat and can swiftly culminate in multiple organ failure [33]. Obstruction of the microcirculation is intrinsic to the pathophysiology of severe malaria [34], with factors such as sequestration, resetting [35], and diminished RBC deformability contributing to impaired microcirculatory flow. The introduction of hemozoin—a byproduct formed from the digestion of blood by some blood-feeding parasites that are generated abundantly during malaria infection and released into circulation upon schizont rupture—can damage cell membranes via oxidative mechanisms, inducing rigidity [36], This loss of flexibility compromises the ability of RBC to navigate through the microcirculation, significantly exacerbating the severity of malaria.

Diabetes mellitus is associated with hemorheological irregularities [18], such as heightened plasma viscosity, amplified RBC aggregation, and decreased RBC deformability [37]. These abnormalities contribute significantly to the initiation and progression of vascular complications in diabetic patients. Nonenzymatic glycation of various proteins, particularly red cell-membrane glycoproteins and hemoglobin, is commonly observed in patients with diabetes, possibly accounting for the altered rheological properties of erythrocytes [38,39]. Given the metabolic imbalances in diabetes mellitus and their impact on hemorheological properties, reducing plasma viscosity may offer a strategic approach to enhance microcirculation, hence delaying the progression of microangiopathy in diabetic patients [40].

Sepsis initiates from an infection occurring in various anatomical sites such as the lungs, urinary tract, abdomen, or skin, triggering an immune response characterized by the release of inflammatory mediators [24]. In sepsis, this immune response becomes dysregulated, resulting in systemic inflammation that damages blood vessels and increases vascular permeability, leading to fluid leakage into tissues [23], and reduced RBC deformability [22]. Concurrently, the activation of the coagulation system induces disseminated intravascular coagulation, forming microvascular thrombi that impair blood flow and contribute to tissue ischemia. The resulting hypotension, due to both fluid leakage and impaired vascular resistance, culminates in reduced perfusion and oxygen delivery to organs, leading to tissue hypoxia and multi-organ dysfunction, hallmarks of septic shock. Likewise, diabetic patients, given their increased susceptibility to infections [41], may experience further decreases in RBC deformability during sepsis, exacerbating the impairment of microcirculation. Notably, alterations in RBC rheology—encompassing deformability and aggregation—manifest early in Intensive Care Unit (ICU) patients, especially those suffering from sepsis [20,42].

Precipitate ischemic and post-ischemic reperfusion injuries could resulted from various types of blood vessel occlusions, the severity of which depends on the duration and extent of the occlusion. Numerous experimental models have demonstrated a significant decline in the rheological properties of RBC following ischemia. Hemorheological parameters, such as blood viscosity, plasma viscosity, RBC aggregation, and RBC deformability [25,26], can all be affected during ischemia and reperfusion [43], along with their determining and influencing factors.

In conditions discussed above, storage lesions, sickle cell disease, malaria, diabetes mellitus, sepsis and ischemia, an evident increase in RBC rigidity is observed. For example, in patients with diabetes, early deterioration in RBC deformability is detectable even when renal function appears normal [44,45,46,47]. Moreover, a persistent rise in the impairment of RBC deformability correlates with eventual renal function loss, underscoring the potential of RBC deformability as an early biomarker for disease progression in these patients [48,49].

Furthermore, a decrease in RBC deformability may sometimes herald more severe, and often irreversible, pathological transformations in other essential organs and organ systems. In certain scenarios, it may even precipitate organ injury. For instance, patients with hemoglobinopathies often exhibit elevated tricuspid regurgitant velocity and pulmonary hypertension, particularly those with a history of renal or cardiovascular complications, increased systemic systolic blood pressure, and abnormalities in markers of hemolytic anemia, such as anemia, reticulocytosis, increased lactate dehydrogenase, aspartate aminotransferase, and bilirubin levels. Additional factors like iron overload, cholestatic liver dysfunction (elevated alkaline phosphatase), renal insufficiency, further exacerbate these conditions [50,51,52,53,54]. Given the profound correlation between RBC deformability and various diseases, a deeper understanding of this feature could facilitate the development of more accurate and effective diagnostic and therapeutic strategies in clinical practices dealing with RBC-related disorders.

Additionally, managing prevalent chronic diseases in the general population—such as diabetes mellitus and sickle cell disease—could potentially be beneficial. Therefore, investing in research and understanding RBC deformability could yield more integrated healthcare approaches and improved patient outcomes.

## 3. Mechanics Evaluation of RBC Deformability

From a classical mechanics perspective, an RBC can be considered a soft material characterized by viscoelastic properties. According to the Kelvin–Voigt model, the elasticity represents the energy-storing properties, while the viscosity embodies the energy-dissipating properties.

### 3.1. Elasticity

Elasticity characterizes the RBC resistance to deformation, which is explained by three fundamental modes:

(1) Area expansion/compressibility modulus (K): it is mainly domain by the RBC membrane [55]. Moreover, different from the other modulus, it corresponds to the surface dilation or so call expansion with shear or bending.

$$T_{t}=K\frac{∆A}{A_{o}},$$

where $T_{t},\;A_{o}$ and **∆A** correspond to the isotopic tensile force, the original surface area, and the increase in surface area, respectively.

(2) Shear elastic modulus (μ): elongation or shear of the surface area that contributes from the spectrin network [56].

$$T_{s}=\frac{\mu }{2}(\lambda^{2}-\lambda^{-2})$$

where $T_{s}$ is the shear force and λ is the extension ratio.

(3) Bending modulus (**B**): represents the energy needed to change the surface curvature [57].

$$M=B(C_{1}+C_{2}-C_{3})$$

where M is the bending moment. $C_{1}$ and $C_{2}$ are two principle curvatures, and $C_{3}$ is the curvature in the stress-free state.

### 3.2. Viscosity

Viscosity is the property for RBC to resist the rate of deformation. The viscosity of RBC is determined by the membrane and cytoplasm viscosity.

(1) Cytoplasm viscosity: normally determined by Hb concentration, which can be measured by dynamic fluctuations of RBC membrane, obtaining 2–5 mPa∙s [58].
(2) Membrane viscosity, considering a 2D condition. $\eta_{2D}$, the coefficient of surface viscosity. The shear force can be expressed as,

$$T_{s}=\frac{\mu }{2}\left(\lambda^{2}-\lambda^{-2}\right)+{2\eta }_{2D}\frac{\partial ln\lambda }{\partial t},$$

where t is the time. In transient conditions, such as RBC recovery from a deformation state, the relaxation constant, $t_{c}$, is provided by the ratio of the elastic shear modulus and the membrane surface viscosity. This ratio indicates that internal friction impedes the recovery of RBC deformation, represented as $t_{c}=\eta_{2D}/\mu$ [59].

### 3.3. Viscoelastic Models and Experimental Methods

RBC exhibit viscoelastic behavior, meaning that the mechanical response combines both elastic and viscous characteristics. At short time scales, the deformation is dominated by elastic response, whereas at longer time scales viscous flow becomes significant, resulting in time-dependent strain recovery and stress decay.

**Figure 2:**
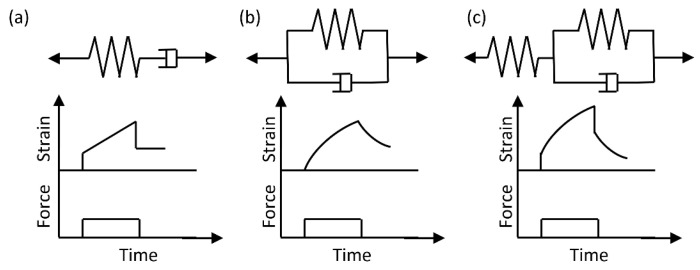
Model for viscoelasticity evaluation. (**a**) Kelvin–Voigt model. (**b**) Maxwell model (**c**) Standard Linear Solid model.

The Kelvin–Voigt model (spring and dashpot in parallel) captures creep by predicting strain that asymptotically approaches equilibrium under constant stress. Conversely, the Maxwell model (spring and dashpot in series) describes stress relaxation through exponential stress decay under constant strain. For more complex biological responses, the Standard Linear Solid model (combining Kelvin–Voigt and Maxwell elements) integrates instantaneous elasticity, delayed recovery, and stress relaxation, offering a holistic representation of RBC mechanics (Corresponding models are illustrated in Figure 2).

Experimentally, creep test apply a constant stress and record strain as a function of time, validating Kelvin–Voigt predictions, where J(t) is creep compliance,

$$\varepsilon \left(t\right)=J(t)\sigma ;$$

σ denotes stress while ε denotes strain. Stress relaxation test apply a constant strain and record stress decay over time, validating Maxwell models, where $G\left(t\right)$ is called the shear modulus or relaxation modulus.

$$\sigma \left(t\right)=G(t)\varepsilon ;$$

Dynamic testing apply oscillatory stress or strain over a range of frequencies to obtain storage modulus G′ and loss modulus G″, quantifying the elastic and viscous contributions.

Additionally, the deformation index, representing the deformation of RBC and providing a general method to demonstrate the viscoelastic properties of RBC, is given by (L + W)/(L − W). Here, L and W denote the major and minor axes of the deformed ellipsoid, respectively [60]. The discussed mechanical parameters, describing the viscoelastic properties of RBC and the techniques that can measure them, are listed in Table 2 [61] (☑ indicates the technique can measure the corresponding parameter).

## 4. Techniques for Measuring RBC Deformability

Measurement techniques for analyzing RBC deformability fall into three categories. The first category, aimed at individual RBC, includes methods such as (1) micropipette aspiration, (2) optical tweezers, (3) atomic force microscopy, (4) magnetic twisting cytometry, and (5) quantitative phase imaging. The second category, focusing on blood rheology, employs bulk-measurement techniques like (1) blood viscometry and ektacytometry, (2) filtration tests, and (3) Erythrocyte Sedimentation Rate. The third category introduces the advanced microfluidic method including (1) hydrodynamic stretching, (2) passing through constrictions, (3) motion and shape analysis, and (4) image and machine learning technique. (summarized in Table 3).

It is essential to recognize that RBC deformability is a complex characteristic influenced by the interplay of multiple factors. These include the surface area-to-volume ratio, cytoplasmic viscosity, and the viscoelastic properties of the cell membrane. Each of these variables is uniquely influenced by various pathophysiological processes and by the different measurement techniques used. Consequently, each technique evaluates cell deformability based on specific criteria and may capture only certain aspects of cell deformability behavior, highlighting the inherent limitations of each technique [91].

### 4.1. Single Cell Measurement Techniques

#### 4.1.1. Micropipette Aspiration

**Figure 3:**
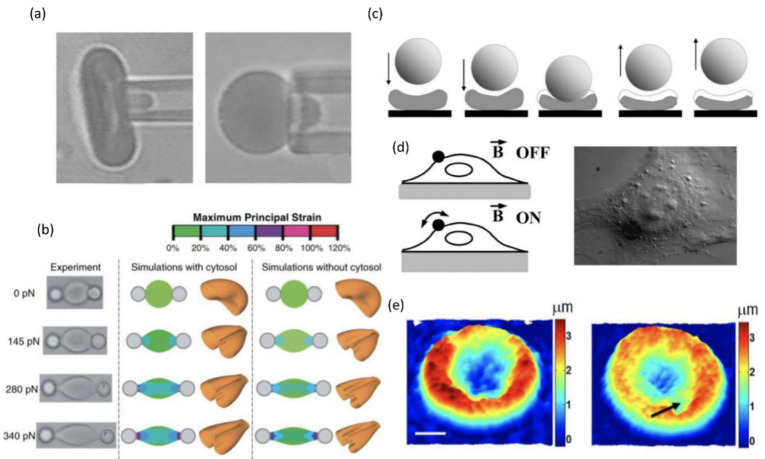
Methods for individual RBC deformability measurement. (**a**) Micropipette aspiration with a flaccid and swollen RBC. Figure adapted from [93] with permission. (**b**) Optical tweezers stretched red blood cell from 0 to 340 pN. Figure adapted from [95] with permission. (**c**) Atomic force microscopy contacting the sample, causing deformation and relaxation. Figure adapted from [85] with permission. (**d**) Magnetic twisting cytometry schematic and micrograph of a cell with a bead attached on its top. Figure adapted from [96] with permission. (**e**) Quantitative phase imaging for health and ring stage RBC. Figure adapted from [97] with Copyright 2008 National Academy of Science.

Micropipette aspiration operates on the principle that the volume of aspirated cells hinges on their mechanical properties, with key parameters being the pressure required to aspirate a distance equivalent to the pipette radius and the ratio of aspirated membrane length to pipette radius at given pressure. The shear modulus is inferred from deformation length (Figure 3a) [92,93]. This technique involves preparing a glass micropipette (1–3 μm inner diameter) under microscopy to target RBC, applying negative suction pressure (10 pN to 100 nN) for aspiration, where aspirated volume depends on RBC viscoelasticity. Measurements include estimating aspiration pressure when deformation equals pipette radius [92], determining membrane length-to-radius ratios, gauging pressure for full-cell aspiration [76], calculating area expansion, and assessing shear modulus via $\frac{D_{p}}{R_{p}}\propto \frac{PR_{p}}{\mu }$ where $D_{p}$ denotes aspirated length, $R_{p}$ denotes pipette radius, P is pressure, μ is elasticity coefficient [94].

Advantages encompass straightforward, economical implementation with piconewton force resolution and capacity for bulk cell examination. However, it requires meticulous vacuum control and imaging coordination, suffers from micron-scale spatial resolution limitations, risks cell damage during deformation, and exhibits pipette-size dependency. Smaller pipettes (<1.0 μm) emphasize membrane shear modulus effects while larger ones amplify cytoplasmic viscosity and reduced surface-area-to-volume ratio impacts. Additional challenges include friction estimation at pipette-cell interfaces and potential cytoplasm alteration from membrane damage.

#### 4.1.2. Optical Tweezer

Optical tweezers involve a pair of laser beams which transfer momentum and force to capture cells, bacteria, viruses, subcellular organelles, and even DNA strands [98,99]. The momentum shift results from optical refraction at the cellular sample. Typically, silica and polystyrene beads, attached to the cell boundary, are employed to cause a momentum shift due to their high refractive index. The laser entraps particles that are smaller than its wavelength, a phenomenon known as Rayleigh scattering. The laser is navigated along the optical axis to exert opposing forces. The assessment of force and the resultant measurement of membrane stretch distance yield valuable indicators of cellular deformability, including changes in particle volume during compression and subsequent relaxation. It is possible to study both elastic and viscoelastic properties through force versus displacement graphs. The setup comprises an inverted microscope, laser generator, optical mirrors, lenses, and a high-resolution camera with a frame rate of 100–150 fps, suitable for image processing (Figure 3b) [95].

Optical tweezers offer non-invasive, precise manipulation or force measurement of microscale particles (cells, viruses, etc.) across physics, chemistry and biology, avoiding physical contact issues and enabling complex loading via beads or multiple beams. Yet they are prone to optical perturbations (demanding strict alignment), depend on particle or medium refractive indices (limiting experiments to aqueous solutions), handle few particles at once (low throughput), and risk heating or photodamage with prolonged intense laser exposure.

#### 4.1.3. Atomic Force Microscopy

Atomic force microscopy (AFM) works by poking the cell membrane with a probe to measure the forces acting on it (Figure 3c). Its methodology involves directing a laser at a cantilever—equipped with an attached probe—such that the laser reflects toward a photodetector. During interaction between the probe and the cell sample, Van Der Waals forces are the primary influencing forces [100]. By analyzing these interaction segments, researchers can determine various chemical and mechanical properties of the cell, including adhesion, elasticity, hardness, and bond rupture lengths. Currently, AFM has proven instrumental in studying RBC deformability in conditions like thalassemia, diabetes, and sickle cell disease [85,101,102,103,104].

AFM offers advantages such as detecting piconewton-level forces; investigating nanoscale subcellular structures (membranes or nuclei) [102,104]; providing detailed data on specific cell points, e.g., such as Yong’s modulus, elasticity, Hamaker constant, adhesion and surface charge densities [105]; enabling quantitative analysis of local membrane nanomechanics; and generating 3D surface profiles. However, it has drawbacks such as limited ability to probe multiple cell points with high temporal resolution, risk of overestimating force-indentation curves due to unintended membrane deformation, tip shape or attachment altering force-deformation curves reducing result transferability, and reliance on skilled technicians with time-consuming operations.

#### 4.1.4. Magnetic Twisting Cytometry

Magnetic Twisting Cytometry (MTC) works by applying both static and oscillating magnetic fields to ferromagnetic microbeads, which are attached to a cell membrane. The applied magnetic fields cause these microbeads to undergo both rotational and translational movements, leading to torques being applied to the cell membrane (Figure 3d) [96,106]. First, a CCD camera captures membrane bead movements, displacement from torque is calculated, then membrane stiffness and loss modulus are derived; by adjusting magnetic force frequency or magnitude, these properties are measured across driving frequencies, with applications like studying malaria infected RBC stiffness at febrile temperature (41 °C) [107].

MTC advantages include minimal biological specimen heat and photodamage, broad media compatibility due to low magnetic susceptibility of cells and fluids, ability to apply constant force without feedback loops, and flexible bead attachment. However, drawbacks involve non-uniform bead-induced stress on samples, variability in bead population magnetic torques, inability to eliminate bead torque, and video-based detection resolution limits for micro-scale beads.

#### 4.1.5. Quantitative Phase Imaging

Quantitative Phase Imaging (QPI) leverages the principle of laser interference to measure the optical phase changes, which is captured as amplitude and phase images (Figure 3e). It has been applied to measure the spatiotemporal coherence, shear modulus, and viscoelastic properties of RBC [97,108,109].

QPI offers several advantages for RBC studies. First, it measures dynamic membrane fluctuations, calculates cellular dry mass [110], aids in studying how factors like osmotic pressure and malarial infection affect RBC deformability [111], enables simultaneous measurement of cytoplasmic hemoglobin (Hb) concentration (derived from reconstructed reflected index distribution [112]) and cytoplasmic viscosity, and is accessible and cost-effective for investigating conditions like sickle cell anemia [113]. However, it has drawbacks: it is less suitable for thick specimens, where light scattering and refractive index variations can distort images; additionally, reduced contrast from center to edges of larger objects (shade-off) and bright areas surrounding specimens can obscure details (halo effects), particularly affecting RBC morphology assessment and introducing artifacts in image analysis [111].

### 4.2. Bulk Measurement Techniques

#### 4.2.1. Blood Viscometer and Ektacytometry

**Figure 4:**
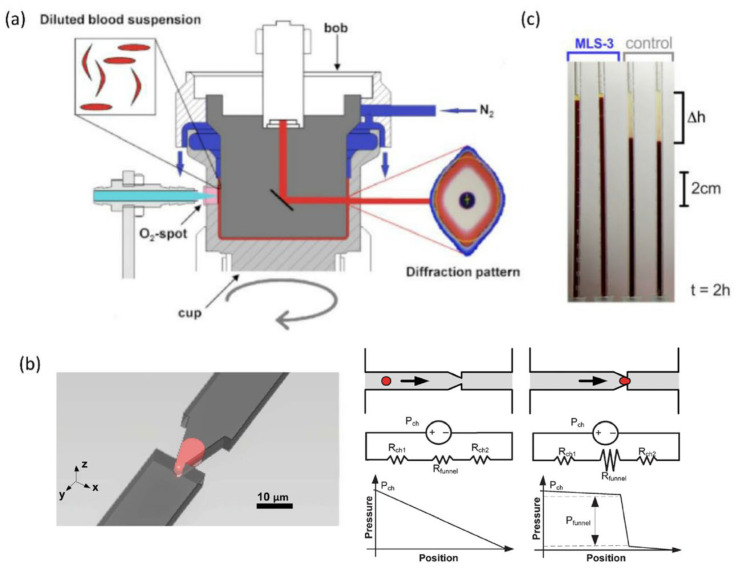
Bulk measurement techniques. (**a**) Schematic setup of the ektacytometer. Figure adapted from [88] with permission. (**b**) A schematic picture of a single cell being deformed through a pointed constriction (left). Pressure profiles of a microchannel containing a free-flowing cell and a cell constrained in the constriction (right). Figure adapted from [118] with permission. (**c**) Standard Westergren tubes were filled with full blood and left to rest for 2 h. Figure adapted from [119] with permission.

Conventional rotational blood viscometers work by detecting the torque needed to spin an object (like a disk or cylinder) in fluid against viscous resistance, with rotation speed directly relating to fluid viscosity (Figure 4a). Stress-controlled viscometers use constant torque while monitoring rotation speed, whereas rate-controlled systems maintain steady speed and measure applied torque; they are classified by shape (cylinder, cone plate, parallel plate) based on their components. The ektacytometer, combining laser diffraction and viscometry, measures RBC deformability, which applies controlled shear stress to RBC via a rotating outer cylinder and stationary inner cylinder, and infers deformability from laser diffraction patterns produced by ellipsoid-like deformed RBC [114]. Commercial versions like LORCA, Rheodyn SSD, and RheoScan-D exist, with studies showing they offer acceptable precision for detecting reduced RBC deformability [88,89]. And the method has found applications in studying RBC deformability in several hemolytic disorders, such as hereditary pyropoikilocytosis, hereditary spherocytosis, and hemoglobin C-C [18,115,116,117].

The calculation of torque, viscosity and velocity are straightforward in Barber’s report [120]. Additionally, for ektacytometer, the degree of RBC deformation is estimated from the elasticity of the diffraction pattern as:

$$EI=\frac{L-W}{L+W}.$$

where EI is the elongation index, while L and W denote the long and short axis of the diffraction pattern [121].

Ektacytometers and blood viscometers have several advantages, that they are simple, effective, and widely adopted, specifically measuring RBC elongation under defined shear stress in a viscous environment; viscometers can measure blood viscosity across a wide range of shear rates, with applied shear stress being homogeneous. However, they have drawbacks that standard shear devices are large and cumbersome, often require substantial reagents, and lack sophisticated control over the mechanical environment experienced by the cell.

#### 4.2.2. Filtration Test

The filtration test revolves around the concept of analyzing RBC behavior as they navigate through filtration prepared with holes ranging from 3–5 μm in diameter (Figure 4b) [118,122,123]. This method induces a negative pressure to encourage whole blood to pass through a membrane filter dotted with 3–5 μm holes. The deformability of RBC is directly linked to the rate of flow through these pores. Either the flow duration or the volume of blood filtered over a fixed period (approximately 1 min) can be used to compute the RBC deformability. The RBC deformability index (DI) in unit time can be given by: DI $=\frac{V\times hct}{time}$. (Where hct is the hematocrit of the sample) [29]. This method has been utilized in various studies focusing on RBC deformability, including the effects of RBC storage, diabetes, sepsis, sickle cell disease, and homozygous β thalassemia [29,41,124,125].

Filtration offers advantages such as being independent of shear stress variability; being highly responsive to reductions in the surface area to volume ratio based on filter pore size; and being widely applied due to simple instrumentation, clinical relevance, and high reproducibility. However, it has drawbacks: lower sensitivity to increased cytoplasmic viscosity; it only measures the ability of RBC to undergo folding deformations (critical for entering narrow capillaries); unlike micropipette aspiration, it cannot be applied to individual cells or under defined stress conditions; and it suffers from leukocyte clogging.

#### 4.2.3. Erythrocyte Sedimentation Rate

The Erythrocyte Sedimentation Rate (ESR), a common clinical hematology test, measures how quickly RBC settle at the bottom of a test tube over a specified period, based on the principle that RBC tend to form rouleaux stacks, accelerating their settling (Figure 4c) [119]. The technique involves placing whole blood mixed with an anticoagulant into a standard tube (e.g., Westergren or Wintrobe), positioning it vertically, and leaving it undisturbed—typically for an hour. Normal ESR values vary by age and sex (e.g., males < 50: ≤15 mm/h; females < 50: ≤20 mm/h, etc.) [126], with elevated levels potentially indicating underlying illnesses like anemia, infections, or kidney disease [127]. Notably, this long-established test has recently been used as an indicator of RBC deformability [119,128] and serves as a potential marker for conditions such as Chorea-acanthocytosis, a rare neurodegenerative disease characterized by deformed acanthocytes.

ESR test offers advantages such as being easy to perform and cost-effective, and helps monitor the progression or remission of diseases like rheumatoid arthritis and temporal arteritis. However, the results can be affected by factors such as age, sex, pregnancy, anemia, and certain medications, leading to potential false positives or negatives; ESR may not reflect acute changes in a patient condition quickly, limiting its utility in emergency settings; it cannot be used as a sole diagnostic tool for specific diseases, as many different conditions can cause an elevated ESR.

### 4.3. Microfluidic Methods

#### 4.3.1. Hydrodynamic Stretching

**Figure 5:**
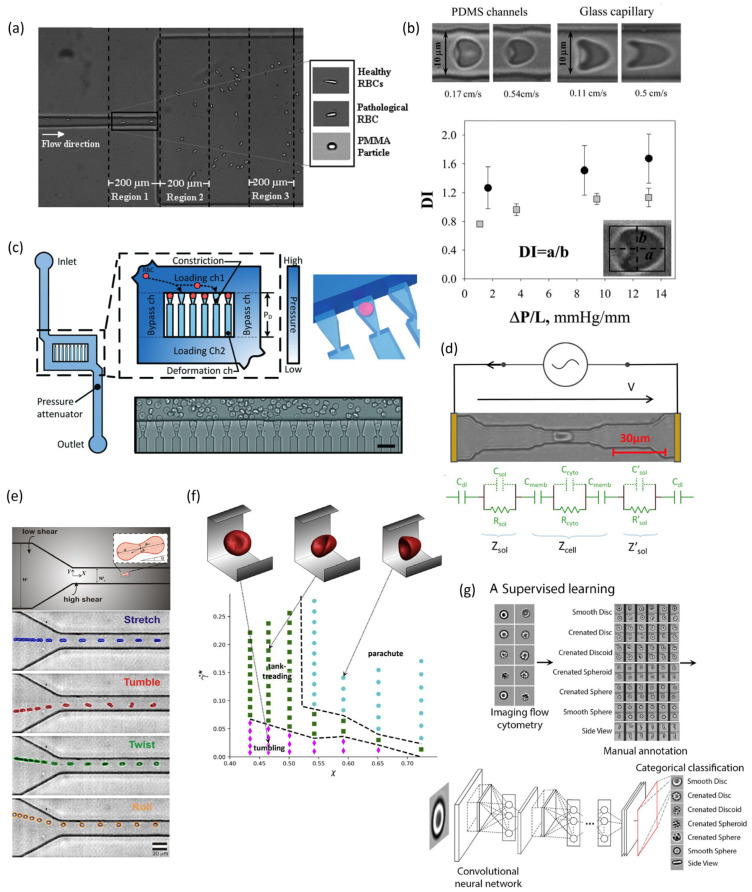
Microfluidic-based measurement techniques. (**a**) Hydrodynamic stretching by a sudden expansion. Figure adapted from [78] with permission. (**b**) Deformability index study for RBC parachute shape. Figure adapted from [74] with permission. (**c**) Constriction method of array design. Figure adapted from [133] with permission. (**d**) Constriction method of impedance sensing. Figure adapted from [134] under Creative Commons CC BY license. (**e**). Motion analyses. Figure adapted from [135] with permission. (**f**) Shape analysis. Figure adapted from [136] with permission. (**g**). Image and machine learning technique. Figure adapted from [137] under Creative Commons Attribution-Non-commercial-NoDerivatives License 4.0 (CC BY-NC-ND).

Microfluidic channel design operates on the principle of manipulating hydrodynamic forces to deform cells as they flow through the channels, with cell deformation typically recorded by a camera. The hydrodynamic methods employed in such microfluidic devices can be broadly categorized into two types: pressure drag dominant and shear stress dominant. The pressure drag dominant method utilizes the momentum of high-speed flow to create a stagnation point within a specifically designed channel, where the impinging flow induces cell deformation [129]. On the other hand, viscoelastic fluids are incorporated to generate shear stress, thereby offsetting the need for high flow velocities [130]. However, this approach is less frequently applied to RBC deformation due to the complex shape and orientation of RBC, with only limited studies—such as numerical simulations using cross-slot channels—having explored its utility [131]. In contrast, the shear stress dominant method, which is more widely used for RBC stretching, involves the use of narrow channels and viscous fluids to increase the shear stress exerted on cells [132]. This approach employs two primary techniques: adding viscoelastic fluids to stretch RBC into an ellipsoid-like shape (Figure 5a) [78] and deforming RBC into a parachute-like shape, followed by measuring the deformation (Figure 5b) [74,90].

Microfluidic devices offer several advantages: their non-contact working principle avoids the effects of surface friction or size correlation; they enable precise control and manipulation of small fluid volumes for highly accurate experiments, while saving space, labor, and measurement time compared to traditional methods; they can be repeatedly fabricated to ensure consistent and reproducible results; they allow cells to be exposed to pulsatile or chaotic flow, mimicking physiological conditions, and require small sample and reagent volumes, reducing the consumption of valuable materials; additionally, their design can closely reflect in vivo physiological conditions.

However, they have limitations: due to the small size of RBC, imaging requires high magnification (e.g., 20× or 40×) to capture clear, dynamic deformations at high speeds, which may restrict the field of view or challenge high-speed cameras in maintaining clarity while tracking fast-moving RBC; the use of additional viscoelastic fluids to facilitate RBC deformation may affect the fragility of cell, causing discomfort, damage, or altering their behavior and deformability; furthermore, the complex shapes and varied orientations of RBC as they enter microchannels make it difficult to control their positioning and ensure a consistent parachute-like shape, hindering accurate and reliable measurements due to inherent variability in RBC properties and behaviors.

#### 4.3.2. Passing Through Constrictions

The principle of this microfluidic approach is similar to the filtration method, measuring the ability of RBC to squeeze through small constrictions, with microfluidics integrating the filtration concept with multiple techniques. Its techniques include: constriction arrays, where multiple channel constrictions enhance cell processing throughput, with RBC injection controlled by constant pressure and deformation recorded via imaging (Figure 5c) [133,138]; and single constrictions, where RBC stiffness is indicated by transit time and velocity through the constriction, detected via imaging [139], pressure drop [140], or impedance (Figure 5d) [134]. This method has proven effective in applications such as sepsis [141], malaria [133], sickle cell disease, and hereditary spherocytosis [142].

This method offers notable advantages, such as constriction arrays that significantly boost measurement throughput while enabling single-cell characterization instead of bulk analysis; it can also integrate multiple techniques (e.g., electrics, optics, acoustics), allowing pressure or impedance detection to replace imaging—simplifying cumbersome image processing and avoiding challenges related to image quality requirements for small cells. Additionally, its straightforward working principle and simple system setup facilitate commercialization. However, the constriction method is prone to clogging during experiments; its contact-based working principle means measurement results are heavily influenced by cell size and surface friction [143,144,145]; and clogging also renders the device non-reusable, potentially increasing costs due to the need for disposables.

#### 4.3.3. Motion and Shapes

The principle of this approach lies in the fact that the biconcave, highly deformable RBC exhibit diverse deformed shapes and motions during microchannel migration, which are linked to factors like flow rate [146,147,148], channel confinement [149,150,151], RBC deformability [152,153,154], viscosity ratio [154,155,156,157], and entry orientation [158]. By analyzing these dynamic shapes and motions under controlled conditions, RBC viscosity and deformability can be revealed. The technique involves two key analyses: motion analysis, which identifies four distinct motions (stretching, tumbling, twisting, rolling which shown in Figure 5e [137]) and complex motions in RBC traversing constrictions without prominent deformation [135,159]; and shape analysis, where narrowing microchannel constrictions to 3–13 μm [153] and increasing flow velocity induce deformation, with observed parachute or slipper shapes [147,160] providing insights into RBC mechanical properties and deformability (Figure 5f) [136].

This method offers advantages as a non-invasive approach that avoids additional buffers or invasive techniques, making it convenient and gentle for studying RBC behavior; moreover, by mimicking physiological conditions in microfluidic channels, it can provide valuable insights into RBC movement, interactions, and deformation-related factors relevant to hematology problems. However, it has drawbacks: data analysis is challenging, as tracking RBC migration motion (including orbit changes in flipping cells) is complex for automated methods, often requiring time-consuming manual analysis; despite progress, the complex dynamics of RBC deformation remain incompletely understood, necessitating further research; additionally, real experiments often show numerous undefined transitional shapes between the known parachute and slipper forms [161], which, influenced by factors like flow velocity and channel geometry, complicate consistent shape categorization.

#### 4.3.4. Machine Learning

Different from previous mentioned deformability measuring methods, which involve physically probing RBC or observing dynamic RBC changes in shapes, this section introduces a passive method that focuses on assessing RBC morphology in their equilibrium sate. The morphological changes in RBC are an important indicator for RBC state, which is primarily attributed to membrane deformation resulting from degradation and oxidative damage [162,163].

Machine learning algorithms, such as deep learning, can analyze and identify patterns in the images of RBC, enabling automated recognition and classification of different morphological changes (e.g., created disk shape, spherical cells, crenated ball or smooth ball forms). These models learn from the labeled data, extracting relevant features and building a representation of the different morphological categories (Figure 5g) [137,164].

A recently developed method, IRIS, combines motion and shape analysis with machine learning to study RBC deformability. The first step involves directing RBC into a microchannel where they undergo shape deformation due to the flow conditions. Subsequently, a classification system consisting of six defined shape categories is used to identify the state of each RBC. To automate the shape classification process, the YOLOv5 model [165,166], is to accurately classify RBC into the predefined shape categories. Once the RBC are classified, shape analysis is performed on each group to study their deformability.

The machine learning method significantly reduces human labor, minimizes subjective bias for more objective and consistent results, and provides a comprehensive approach to studying RBC deformability by enabling automated identification and classification of RBC shapes while enhancing the sensitivity of deformability analysis. However, the model training requires large amounts of labeled data; classification results are sensitive to image quality; and model performance depends on training conditions—if encountering unaccounted-for heterogeneity or variations in RBC morphology or conditions, it may produce false results, with limited ability to handle novel scenarios or generalize to new, diverse datasets.

## 5. Discussion and Conclusions

Over the span of several decades, substantial progress in biology and medicine has deepened our understanding of human health, disease processes, and physiological functions. RBC deformability, governed by the surface area-to-volume ratio, cytoplasmic viscosity, and membrane viscoelasticity, is a fundamental determinant of microcirculatory flow and tissue oxygenation. Its impairment has been linked to a broad spectrum of pathological conditions, including hemoglobinopathies, infectious diseases, metabolic disorders, and vascular dysfunctions. In many cases—such as diabetes mellitus or storage lesions in transfused blood—mechanical alterations occur before overt clinical symptoms, underscoring the potential of deformability as an early, non-invasive biomarker for disease progression and therapeutic monitoring. The unique composition of RBC, lacking a nucleus and most organelles, further enhances their suitability for mechanical characterization, as deformability changes can directly reflect alterations in membrane proteins, hemoglobin concentration, and ATP metabolism without molecular labeling.

A wide variety of techniques have been developed to measure RBC deformability, each offering distinct advantages and limitations. Single-cell approaches (e.g., micropipette aspiration, optical tweezers, atomic force microscopy) provide detailed mechanical parameters but are often low-throughput and require specialized instrumentation. Bulk rheological methods (e.g., blood viscometry, ektacytometry, filtration tests) capture population-level properties but obscure cell-to-cell heterogeneity. More recently, microfluidic platforms have emerged as a promising compromise, combining higher throughput with physiologically relevant flow conditions. Within these, constriction-based assays offer notable advantages in throughput, integration flexibility, and cost-effectiveness, but their contact-based nature raises concerns about surface interactions and orientation-dependent variability. Non-contact methods avoid these issues but are often optimized for spherical cells, limiting their applicability to the discoidal RBC.

Despite decades of research, the translation of RBC deformability testing into routine clinical practice remains limited. Major barriers include (i) the absence of universally accepted reference values and insufficient large-scale clinical validation, and (ii) operational complexity or low throughput in most existing systems.

For the future work, integrating motion and shape analysis with artificial intelligence offers a compelling pathway to solve those challenges. RBC display distinct in-flow morphologies and dynamic transitions—such as parachute, slipper, and tumbling states—that are sensitive to subtle changes in membrane elasticity, cytoplasmic viscosity, and surface properties. These morphodynamic features can be captured under physiologically relevant microfluidic flow without chemical labeling, preserving near-native conditions. Coupling such image streams with convolutional neural networks or transformer-based architectures enables rapid, automated classification of thousands of cells per second, matching the throughput of flow cytometry while eliminating subjective bias. Beyond classification, AI models can extract quantitative motion descriptors and map them to biophysical parameters, enabling multidimensional profiling rather than reliance on a single deformability index. Physics-informed and domain-adaptive learning strategies could further enhance robustness across variations in optics, flow rates, and channel geometries, paving the way for standardized, multi-center datasets suitable for clinical validation.

In conclusion, RBC deformability is a versatile and sensitive biophysical marker with broad diagnostic and prognostic value. Realizing its clinical potential will depend on overcoming technical and standardization challenges, validating predictive utility in large, diverse patient cohorts, and integrating engineering innovations—particularly AI-enhanced imaging—into practical, point-of-care devices. Such advances could position deformability testing as a routine tool in personalized medicine and preventive healthcare.

## Figures and Tables

**Table 1:** Summary of Disorder Conditions Affecting RBC Deformability.

Disorder	Pathophysiology	Deformity in Cell	Test to Detect the Disorder
Storage lesions	ATP depletion and calcium accumulation	Viscosity increase	Microfluidic-based method [12]
Sickle cell anemia	Autosomal recessive disease with a mutation in β globin chain. Normal HbA is replaced by HbS. On oxidation stress the abnormal HbS polymerizes to cause sickle cell deformity and leading to sickle cell crisis [13].	Increase in the plasma viscosity. Increase in the aggregation of RBC causing occlusion of the vessels [14].	Electrophoresis [15]
Malaria	Caused by either of the five varieties of single celled parasites named Plasmodium through the bite of female anopheles mosquito.	Loss of discoid shape of the cell. Increase in the rigidity of the membrane. Increased adhesiveness to endothelial surfaces leading to aggregation and obstruction in microcirculatory flow [16].	Blood smears, Rapid antigen detection test [17].
Diabetes mellitus	Type 1 is caused by autoimmune destruction of pancreatic β cells. Type 2 is caused by peripheral resistance to insulin. GDM: hyperglycemia or hyperinsulinemia in utero	Impaired glucose metabolism thereby deforming the RBC membrane. An increase in blood viscosity increases RBC aggregation [18].	Random blood glucose levels. Fasting blood glucose levels. Postprandial blood glucose levels HbA1c levels. GDM: membrane flickering [19]
Sepsis	Infectious insult results in a local inflammatory response which spills over and causes systemic symptoms secondary to various cytokines. It is called systemic inflammatory response syndrome. These cytokines also activate the extrinsic coagulation pathway thereby causing coagulopathy [20].	Changes in microcirculation [21]. Increased RBC aggregation [21,22]. Decrease in RBC and WBC deformability [23]. Loss of capillary density [23]. Change in microvascular reactivity. WBC-endothelial cell adhesion and leaking of the vasculature coagulation disturbances [24].	Blood culture. Coagulation profile. Total blood count. Peripheral smear.
Occlusive disorders	Caused by ischemia secondary to atherosclerosis and post ischemic reperfusion injuries	Increase in RBC aggregation and blood viscosity [25]. Decrease in RBC deformity [26].	Computed tomography. Magnetic resonance angiography.

**Table 2:** Overview Of Measurable Parameters And Corresponding Assessing Techniques.

	Micropipette	Viscometer	Microfluidics	Ektacytometry	AFM	Optical Tweezer
Volume	☑ [62,63,64,65]		☑ [66,67]			
Surfaces area	☑ [62,63,64,65]		☑ [66,67]			
Cytoplasmatic viscosity		☑ [68,69]				
Surface viscosity	☑ [55,70,71,72,73]		☑ [74]	☑ [75]		
Shear elastic modulus	☑ [70,71,72,76,77]		☑ [78]	☑ [75]		☑ [79,80]
Bending modulus	☑ [57,81]				☑ [82]	☑ [83]
Relaxation constant	☑ [70,72,73]	☑ [84]	☑ [74]		☑ [85]	☑ [80]
Area compressibility	☑ [55,71,86]					
Yong’s modulus					☑ [87]	
Deformation index		☑ [88,89]		☑ [74,90]		

**Table 3:** Summary of the Techniques for Measuring RBC Deformability.

Category	Technique	Principle	Key Pros	Key Cons
Section 4.1 Single cell Measurement	Section 4.1.1 Micropipette Aspiration	Deform cell into a constriction	Straightforward and economical	Time-consuming
	Section 4.1.2 Optical Tweezers	Momentum and force of photon	Non-invasive, versatile, and highly accurate	Requirement of optical alignment is pretty high
	Section 4.1.3 Atomic Force Microscopy	Poking the cell membrane with a probe	Can detect forces at the piconewton level and investigate nanoscale cellular structures	Limited in its ability to probe multiple points in a cell
	Section 4.1.4 Magnetic Twisting Cytometry	Apply oscillating magnetic fields	Flexibility in bead attachment	Non-uniform stress
	Section 4.1.5 Quantitative Phase Imaging	Laser interference	Dynamic membrane fluctuations can be observed	Thick specimen, shade-off and halo effect
Section 4.2 Bulk Measurement Techniques	Section 4.2.1 Blood Viscometry and Ektacytometry	Detecting the torque required to spin an object	User-friendly	Require substantial amounts of reagents
	Section 4.2.2 Filtration Tests	Encourage whole blood to pass through a membrane filter	Simplicity of instrumentation	Lower sensitivity to increments in cytoplasmic viscosity
	Section 4.2.3 Erythrocyte Sedimentation Rate	RBC tend to stack into rouleaux and sediment quick	Low cost	Cannot be used as a sole diagnostic tool
Section 4.3 Microfluidic-Based Measurement	Section 4.3.1 Hydrodynamic Stretching	Hydrodynamic forces deform cell	Non-contact working principle	Only suitable for sphere cell
	Section 4.3.2 Passing Through Constrictions	Squeeze through a small constriction	Integrating multiple techniques	Suffer from the clogging issue
	Section 4.3.3 Motion and Shapes	Due to the deformable disk shape interacting with the fluids	Convenient and gentle method	Difficulties in tracking the motion or categorized shapes
	Section 4.3.4 Machine Learning	RBC morphology indicates the biochemical changes	Reduces human labor and minimizes subjective bias	Training of the model requires a large quantity of labeled data

## Abbreviations

RBC	Red blood cell
MCV	Mean Corpuscular Volume
MCHC	Mean Corpuscular Hemoglobin Concentration
ATP	Adenosine Triphosphate
HbS	Abnormal Sickle Hemoglobin
HbA	Normal Hemoglobin
ICU	Intensive Care Unit
AFM	AtomicForce Microscopy
CCD	Charge-Coupled Device
MTC	Magnetic Twisting Cytometry
QPI	Quantitative Phase Imaging
DLS	Dynamic Light Scattering
ESR	Erythrocyte Sedimentation Rate
AI	Artificial Intelligence
GDM	Gestational Diabetes Mellitus
